# P-2174. Politics and Money? Mpox Vaccine Coverage in the US: A Study in Healthcare Variation

**DOI:** 10.1093/ofid/ofae631.2328

**Published:** 2025-01-29

**Authors:** HeeEun Kang, Suzette Rovelsky, Nam Hoon Kang, Elizabeth A Talbot, Richard A Murphy

**Affiliations:** Loyola University Medical Center; White River Junction VA Medical Center, Fairlee, Vermont; Hanshin University, Seoul, Seoul-t'ukpyolsi, Republic of Korea; Geisel School of Medicine at Dartmouth, Lebanon, NH; Dartmouth, Norwich, Vermont

## Abstract

**Background:**

During the United States (US) mpox outbreak of 2022-2023, the federal government distributed mpox vaccines (JYNNEOS) to individual states for distribution to at-risk persons. For local distribution, there was no immediate federal funding available. The vaccines were not commercially available. Vaccination data was reported to states’ public health and the Centers of Disease Control and Prevention. There was considerable variation in vaccine coverage across different states.

**Figure:**
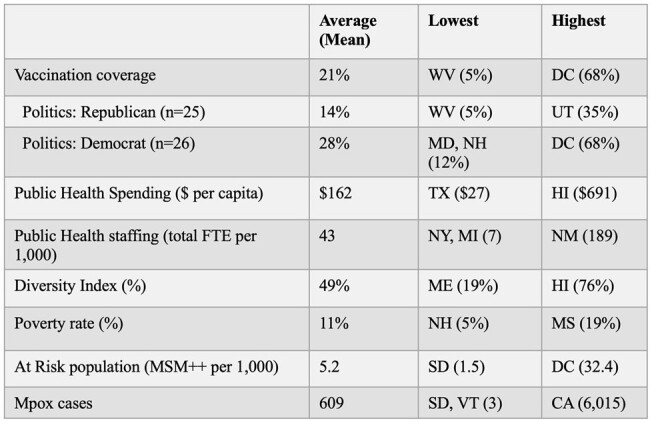

Mpox vaccine coverage and associated factors for US states.

**Methods:**

Using public data, we sought to identify state-level factors associated with full mpox vaccine coverage (two doses), including: political identity, public health spending, public health staffing, diversity index, poverty rate, percentage of population at-risk, and mpox cases. Data sources were: 2020 presidential election result, Association of State and Territorial Health Officials, 2020 US Census, and the CDC. Univariate and multivariable linear regression was performed using all studied variables. P-value of < 0.05 was used to note statistical significance.

**Figure:**
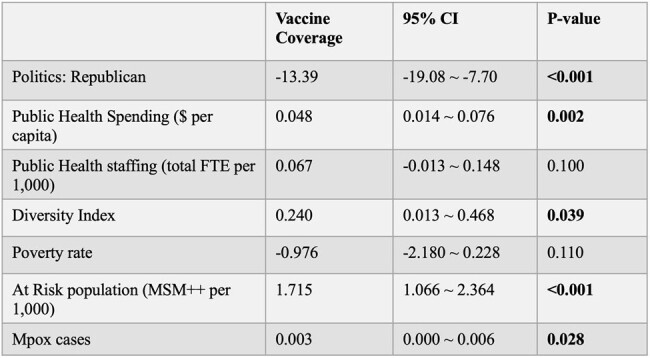

Factors associated with mpox vaccine coverage: univariate linear regression.

**Results:**

Nationally, average full mpox vaccine coverage for at-risk persons was 21%. Univariate analysis showed that Republican political status was associated with lower JYNNEOS vaccine coverage. Higher coverage was associated with higher public health spending per capita, increased diversity index, higher proportion of at-risk population, and greater number of absolute mpox cases. Multivariable analysis showed the following: Republican states achieved -5.65% lower vaccine coverage *(p*=0.036), each $1 increase in public health spending increased vaccine coverage by 0.05% (*p*=0.000), each additional MSM++ (men who have sex with men [MSM] on HIV pre-exposure prophylaxis plus MSM with HIV) per 1,000 people increased vaccine coverage by 1.5% (*p*=0.000), and each additional mpox case increased vaccine coverage by 0.003% (*p*=0.032). Multicollinearity check showed low correlation among tested variables.

**Figure:**
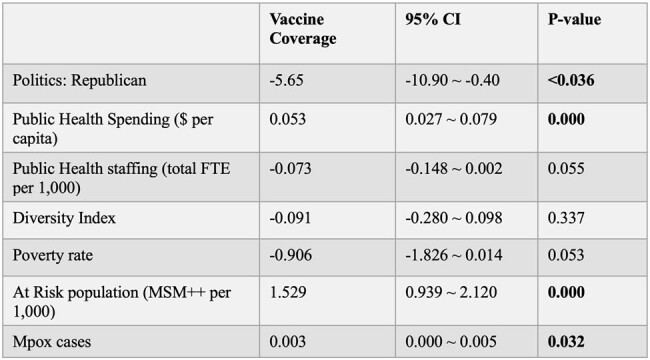

Factors associated with mpox vaccine coverage: multivariable linear regression.

**Conclusion:**

Higher mpox vaccine coverage was achieved in US states with Democratic political orientation, higher public health funding, higher proportion of at-risk population, and higher mpox cases. Additional analyses toward equitable vaccine access are needed.

**Disclosures:**

All Authors: No reported disclosures

